# Woman’s Dress

**Published:** 1890-05

**Authors:** 


### WOMAN'S DRESS.

At the last meeting of the British Association for the Advancement of Science, Mrs. Carmichael Stopes read a paper regarding errors in women's dress, in which she divided the present faults of women's clothing into two classes :—

"1. The temporarily ridiculous or disadvantageous. 2. The permanently injurious. Among the first might be included all the modern forms of crinoline and dress-improver, tied-back skirts, tied-down arms and sleeves, over-long dresses, and over-heavy trimmings, and any absurd fashion that impeded the action, freedom or development of women, or which outraged art. Of the second class, she mentioned first the inequalities and disproportions of clothing, and strongly denounced the use of high heels and tight stays. Fashion blinded the eyes of its votaries. Comparisons she had made of the notes taken by different corset makers, enabled her to say that during the last twenty-five years the female waist had decreased in size by two inches. It was worthy of consideration whether women were justified in following fashion to an extent that not only injured their own health, but tended to lower the physique of a whole nation."

Dr. Kate Lindsay, in a recent lecture said: "When a woman turns her dress into a street sweeper or a carpet sweeper, there should be some sanitary regulation to meet the case. She raises a cloud of dust which settles upon her underclothing and her person. This species of uncleanliness affects both herself and others. Another thing, there is not a little strength wasted in dragging about a heavy, trained skirt. Grace of motion and freedom of action are incompatible with this unneccessary burden. It is well adapted to the affectation of listlessness and languor. But it is to be hoped that our civilization is advancing so rapidly that the time is not far distant when society women will dispense with this 'caudal appendage' as entirely as have the noble army of women who have entered the ranks of workers. Improper dress has not a little influence in retarding the golden dawn of universal suffrage."

